# Alternative Splicing of MXD3 and Its Regulation of MXD3 Levels in Glioblastoma

**DOI:** 10.3389/fmolb.2019.00005

**Published:** 2019-02-19

**Authors:** Tin Ngo, Abraham Corrales, Traci Bourne, Samir Elmojahid, Kit S. Lam, Elva Díaz

**Affiliations:** ^1^Department of Pharmacology, University of California Davis School of Medicine, Davis, CA, United States; ^2^Department of Biochemistry and Molecular Medicine, University of California Davis School of Medicine, Davis, CA, United States

**Keywords:** MXD3, The Cancer Genome Atlas (TCGA), alternative splicing, glioblastoma, MYC/MAX/MXD network

## Abstract

The transcription factor MXD3 is an atypical member of the MYC/MAX/MXD transcriptional network and has been previously shown to be an important regulator of cell proliferation. MXD3 has been shown to be overexpressed and to be required for medulloblastoma and acute lymphoblastic leukemia cell proliferation. In this study we leveraged datasets from The Cancer Genome Atlas to examine MXD3 across several cancers. We find that MXD3 transcripts are significantly overexpressed in ~72% of the available datasets. The gene itself is not frequently altered, while the promoter appears to be hypomethylated. We examine the possibility that aberrant regulation of the MXD3 message is the cause of abnormal MXD3 expression across cancers. Specifically, we looked at MXD3 alternative splicing in glioblastoma multiforme (GBM) and find notable functional differences between the splice variants. The 3′UTR confers differential message stability. Furthermore, the different coding sequences lead to different protein stabilities and localizations. Altogether, these data extend our knowledge of MXD3 in the context of human cancers while characterizing a previously unstudied splice variant of MXD3.

## Introduction

MXD3 is a basic-helix-loop-helix leucine zipper transcription factor belonging to the MYC/MAX/MXD transcriptional network (Grandori et al., 2000). Within this network, the MYC and MXD families compete for heterodimerization with the central cofactor MAX in order to achieve DNA binding at E-box promoter sequences (Ayer et al., 1993). These two families enact opposing transcriptional functions with MYC-MAX heterodimers recruiting transcriptional co-activators leading to the transcription of genes promoting proliferation (Kretzner et al., 1992). In contrast, MXD-MAX heterodimers recruit transcriptional repressors leading to the repression of genes promoting differentiation (Ayer et al., 1996).

However, MXD3 is an atypical member of the MXD family because it behaves more like MYC rather than its MXD relatives (Yun et al., 2007). MXD family members are expressed at various stages of differentiation (Quéva et al., 1998), with the exception of MXD3 which is expressed during the S-phase of the cell cycle (Hurlin et al., 1995; Fox and Wright, 2001; Quéva et al., 2001; Yun et al., 2007). Furthermore, MXD3 has been shown to be upregulated in proliferating cerebellar granule neuron precursors (GNPs), in cerebellar tumors derived from the patched heterozygous mouse model of medulloblastoma (Yun et al., 2007), in human medulloblastoma (Barisone et al., 2012), and in human acute lymphoblastic leukemia (ALL) (Barisone et al., 2015).

Our lab previously found MXD3 to be expressed in a variety of cancers using an online bioinformatics platform to query serial analysis of gene expression (SAGE) cancer datasets (Barisone et al., 2008). SAGE is limited to the number and lengths of sequencing reads (Velculescu et al., 1995). As a result, it is difficult to quantitatively answer how much MXD3 is overexpressed in cancers relative to normal tissue. With the advent of new sequencing technologies, RNA-seq is a more effective tool to investigate gene expression. Moreover, new initiatives to characterize cancer biology provide more comprehensive datasets for researchers. For example, The Cancer Genome Atlas (TCGA) is a consortium effort between the National Cancer Institute and National Human Genome Research Institute. Their goal is to multi-dimensionally classify and characterize cancer using available technologies and make them available to the public. Several platforms to visualize the large amount of available data have been developed by different groups. One such platform is the UCSC Cancer Browser, which allows users to quickly visualize TCGA datasets by chromosome(s) or by gene(s) (Zhu et al., 2009; Sanborn et al., 2011; Goldman et al., 2013). Furthermore, the cBioPortal for Cancer Genomics (Cerami et al., 2012; Gao et al., 2013) provides several tools to visualize multidimensional TCGA sequencing datasets. Additionally, TCGA Wanderer was recently developed to look at TCGA methylation data (Díez-Villanueva et al., 2015). With these tools, scientists can quickly query TCGA data in order to advance their research. Here we use these tools along with our own analyses to answer questions regarding MXD3's putative role in cancers through the analyses of currently available TCGA datasets.

In previous studies across multiple models, it has been shown that both knockdown and persistent overexpression of MXD3 leads to decreased proliferation (Yun et al., 2007; Barisone et al., 2008, 2012, 2015; Ngo et al., 2014). Furthermore, increased MXD3 overexpression directly correlates with decreased cell proliferation in stably expressing MXD3 cell lines (Barisone et al., 2012). Moreover, we showed that acute MXD3 activation results in a transient increase in cell proliferation while persistent activation of MXD3 eventually results in an overall decrease in cell number (Ngo et al., 2014), suggesting that the time course of MXD3 expression dictates the cellular outcome. These results indicate the importance of MXD3 cellular concentrations and time course to the state of the cell.

With the continual updates to available sequenced cDNAs, it recently came to our attention that the MXD3 gene is alternatively spliced at the terminal exon. The new splice variant is likely the result of alternative polyadenylation (APA) of the MXD3 transcript. Previous studies of other genes that undergo APA have suggested that the splice scheme is a mechanism in which cells can regulate transcript levels (Di Giammartino et al., 2011; Shi, 2012) and/or provide further complexity through protein isoforms (Takagaki et al., 1996). Here we leverage available TCGA datasets and examine both splice variants of MXD3 in human glioblastoma cells to investigate the role of MXD3 splicing.

## Materials and Methods

### Cell Culture

Human glioblastoma cell lines, U87-MG and T98G (both from ATCC), were cultured in a standard humidified incubator (5% CO_2_, 37°C) with media consisting of Eagle's Minimum Essential Media (ATCC) supplemented with 10% Fetal Bovine Serum (FBS, Invitrogen). Transfection experiments were performed using either Lipofectamine 3000 (Invitrogen) or Optifect (Invitrogen) according to manufacturer's recommended protocols.

### Constructs

MXD3 constructs were generated in pHM6 (Roche) to create N-terminal HA-tagged proteins. HA-tagged MXD3.E6 (coding sequence only) was previously generated (Barisone et al., 2012). A partial MXD3.E6 cDNA clone containing the 3′UTR of MXD3.E6 was obtained from Origene. A full-length MXD3.E7 cDNA clone was obtained from the National Institute of Technology and Evaluation, Biological Resource Center (NBRC). The 3′UTR sequence of MXD3.E6 and the coding sequence and 3′UTR of MXD3.E7 were obtained from these cDNA clones. Desired sequences were amplified by PCR with primers containing restriction sites for ligation into pHM6. Mutant MXD3 constructs were made using Stratagene's Site Directed Mutagenesis XL kit according to manufacturer's recommended protocols. Primers for site-directed mutagenesis were designed using Stratagene's online primer design platform. All constructs were verified by sequencing. Luciferase constructs, pIS0 (#12178) and pIS2 (#12177), were obtained from Addgene.

### Datasets and Online Visualization Tools

Preprocessed level 3 RNA-seq TCGA datasets were obtained from the UCSC Cancer Genomics Browser (Zhu et al., 2009; Sanborn et al., 2011; Goldman et al., 2013) and directly from the TCGA data portal. The cBioPortal for Cancer Genomics platform was used to visualize MXD3 alteration frequencies across all TCGA datasets (Cerami et al., 2012; Gao et al., 2013). miRNA binding sites were predicted using miRWalk 2.0 (Dweep and Gretz, 2015). Phosphorylation sites were predicted using NetPhos 2.0 (Blom et al., 1999).

### Immunocytochemistry

Cells were grown on 0.01% poly (L)-lysine (Promega) coated glass coverslips in 6-well plates. Upon collection, cells were washed once with phosphate buffered saline (PBS) and then fixed with 4% paraformaldehyde (Thermo Scientific Pierce). Coverslips were then blocked in 5% normal goat serum (Cell Signaling) with 0.01% Triton X-100 in PBS. Subsequently, coverslips were then incubated overnight at 4°C with primary antibodies in 1% bovine serum albumin (Sigma) with 0.01% Triton X-100 in PBS. Secondary antibody incubations were performed in the same buffer at room temperature for 2 h. Lastly, coverslips were mounted onto Superfrost Plus microscope slides (Fisher Scientific) using ProLong Gold Antifade mounting media (Cell Signaling) and allowed to cure overnight. Coverslips were sealed using clear nail polish and subsequently imaged using a LSM 710 confocal microscope (Carl Zeiss) with a 63X/1.4 oil-immersion objective, and with constant settings for laser power, photomultiplier gain, and offset between groups. Pinhole was set at 1 A.U. and resolution of 1024 × 1024 pixels was used for all images. The following primary antibodies were used: rat anti-HA (Roche) at 1:500, mouse anti-PML (Santa Cruz Biotechnology) at 1:50, rabbit anti-MXD3 (AbCam ab50729 targets the common N-terminus of MXD3.E6 and MXD3.E7) at 1:500, and mouse anti-MXD3 (NeuroMab 75-250 targets the C-terminus of MXD3.E6) at 1:500. Secondary antibodies were as follows: goat anti-rat-A488 (Invitrogen) at 1:500, goat anti-MouseIgG1-A594 (Invitrogen) at 1:500, and goat anti-rabbit-A488 (Invitrogen) at 1:500. Coverslips were treated with Hoechst 33342 (Cell Signaling) at 1:25,000 in PBS prior to mounting on microscope slides.

### Immunoblotting

Cells were lysed using either 1.5x Laemilli buffer at a concentration of 10,000 cells/μL or standard RIPA buffer supplemented by 23.4 μM Leupeptin (Roche), 6.1 μM Aproptinin (Roche), 14.5 μM Pepstatin A (Roche), 0.1 mM PMSF (Millipore), and 1 mM Sodium Orthandovate. Samples were then boiled at 100°C for 10 minutes. Extracts were loaded at a range of 50,000–150,000 cells or 5–15 μg of protein and separated on 12% acrylamide gels under denaturing and reducing conditions. Gels were transferred onto 0.45 μm nitrocellulose membranes. Blots were developed using LI-COR's Odyssey imaging system. The following primary antibodies were used: rat anti-HA (Roche) at 1:1000 and rat anti-actin (Abcam) at 1:10,000. Secondary antibodies were as follows: goat anti-rat IRDye 800CW (Li-Cor Biosciences) and goat anti-rabbit IRDye 680RD (Li-Cor Biosciences).

### Lambda Phosphatase Treatments

Transfected lysates were extracted using RIPA buffer with the omission of components that inhibit phosphatase (Sodium Orthandovate, EDTA). 5–15 μg of lysates were then treated with lambda phosphatase (New England Biolabs) in 30 μL reaction volumes for 30 min at 30°C. Subsequently, Laemilli buffer was then added to samples and immunoblotting performed as described above.

### Luciferase Assays

Luciferase assays were conducted using clear bottom white plates (Costar), with Promega's Dual-Glo(R) Luciferase Assay System according to manufacturer's recommended protocols following a 24-h incubation period at 37°C post-transfection. Briefly, Dual-Glo® Luciferase was added to each well (100 μL) and incubated at room temperature for 15 min. Firefly luminescence was measured using a M5 SpectraMax plate reader (Molecular Devices). Dual-Glo® Stop & Glo® was added (100 μL per well) to the 96-well plate and incubated for 15 min at room temperature before measuring Renilla luminescence.

### Quantitative Real Time PCR (qPCR)

RNA was extracted from cells using a RNeasy kit (Qiagen), and reverse transcribed using a RNA to cDNA reverse transcriptase kit (Ambion) according to manufacturer's instructions. Subsequently, SYBR green quantitative real time PCR was conducted using a SYBR green mastermix (Applied Biosystems) in 12 μL reactions with 5 μM primers, and 100 ng of cDNA template. Reactions were run on a 7900 real time PCR machine (Applied Biosystems) with the following conditions: (1) 50°C for 2 min (2) 95°C for 10 min (3) 40 cycles of 95°C for 15 s and 60°C for 1 min. Results were then normalized using the standard ΔΔCt method of analysis. Primer sequences used were as follows: MXD3 (total) forward–*GTGAGAGAGAGGCCGAGCAT*, MXD3 (total) reverse–*CTCCTGCGCTTCTCCAGTTC*, MXD3.E6 forward–*CTCAGACCAAGAGGAGCTGGA*, MXD3.E6 reverse–*TGGGTGAGGAACATCATAGCC*, MXD3.E7 forward–*TCAGACCAAGTCTTGCCTAATG*, and MXD3.E7 reverse–*AGTGGAGACAGAACAGCCTCA*.

### Statistical Analysis and Data Processing/Visualization

Data processing, visualization and statistical analyses were conducted in R version 3.4.3 (R Core Team, 2017) with base packages in conjunction with the following additional packages: broom, data.table, dplyr, ggplot2, Hmisc, scales, and tidyr. Statistical significance between TCGA datasets was assessed with Welch's *t*-test. Statistical significance between experimental and control conditions in qPCR and Luciferase assays was assessed with one-way ANOVA and Tukey's HSD *post hoc* test. Significance was defined as *p* < 0.05(^*^), *p* < 0.01(^**^), *p* < 0.001(^***^).

## Results

### MXD3 Is Overexpressed in Many Types of Cancers With Glioblastoma Being One of the Highest

Preprocessed RNA-seq datasets from TCGA were obtained through the UCSC Cancer Genomics Browser. We considered those datasets that included samples from both normal and tumor samples in order to do comparisons between the two states. Each dataset was then mean centered to the average of their respective normal samples and analyzed by Welch's *t*-test. We find that many cancers significantly expressed MXD3 more than two times relative to normal tissues (Table 1). Across the datasets, we find that MXD3 has the highest overexpression in glioblastoma with a fold change of 4.26 relative to normal tissues (*p* = 1.43E-08, Welch's *t*-test) (Table 1). These data represent total MXD3 expression level.

**Table 1:** MXD3 is overexpressed in many types of cancers with glioblastoma being one of the highest.

**Cancer**	**MXD3 fold change expression in primary tumor relative to normal tissue**	**Sample # (Solid tissue normal)**	**Sample # (Primary tumor)**	***p*-value**	**Statistical significance**
Glioblastoma multiforme	4.26	5	154	1.43E-08	^***^
Uterine corpus endometrioid carcinoma	3.09	24	174	3.86E-07	^***^
Liver hepatocellular carcinoma	2.88	50	371	1.65E-38	^***^
Kidney renal clear cell carcinoma	2.71	72	533	2.09E-41	^***^
Breast invasive carcinoma	2.39	113	1095	1.42E-43	^***^
Sarcoma	2.38	2	258	1.67E-01	ns
Kidney renal papillary cell carcinoma	2.38	32	290	1.14E-23	^***^
Lung adenocarcinoma	2.07	58	511	4.38E-28	^***^
Lung cancer	1.97	109	1013	4.81E-43	^***^
Prostate adenocarcinoma	1.93	52	497	3.45E-17	^***^
Lung squamous cell carcinoma	1.87	51	502	2.90E-17	^***^
Rectum adenocarcinoma	1.44	9	94	6.01E-02	ns
Head & neck squamous cell carcinoma	1.32	43	519	1.06E-03	^**^
Colon & rectum adenocarcinoma	1.28	50	380	7.24E-06	^***^
Colon adenocarcinoma	1.26	41	286	2.07E-05	^***^
Thyroid carcinoma	1.12	59	505	1.12E-01	ns
Pancreatic adenocarcinoma	1.07	4	178	8.17E-01	ns
Thymoma	0.90	2	119	9.03E-01	ns

Table summary of MXD3 mRNA expression in TCGA RNA-seq datasets. Shown are the fold change in expression between primary tumor relative to normal tissue, sample numbers, and statistical test results. Datasets were ranked ordered for potential biological relevance by MXD3 expression fold change. Statistical significance was determined by Welch's t-test. Significance,

** p < 0.01,

*** *p < 0.001; ns, not significant*.

### MXD3 Gene Alteration Frequencies Are Low Across TCGA Datasets

A query of MXD3 gene alteration frequencies using cBioPortal for Cancer Genomics reveals that the MXD3 gene is infrequently altered in cancer (Supplementary Figure 1). This portal considers all types of alterations: mutations, amplifications, and deletions. MXD3 mutation frequencies were highest in kidney renal clear cell carcinoma with a gene amplification frequency of 18%. Alteration frequencies in glioblastoma were less than 1% across two datasets (Supplementary Figure 1). These alteration frequencies are relatively low compared to other genes such as TP53 which has alteration frequencies reaching 90% in TCGA Ovarian samples (data not shown).

### The MXD3 Promoter Region Tends to be Hypomethylated Across TCGA Datasets

A query of TCGA Wanderer (Díez-Villanueva et al., 2015) reveals that DNA methylation levels near the MXD3 promoter tend to be hypomethylated in tumor samples relative to normal tissue (Table 2). Of the 14 TCGA datasets with available DNA methylation data from Illumina's Human Methylation 450k microarray platform, 7 datasets show a significant decrease (*p* < 0.001) while 1 dataset shows a significant increase (*p* < 0.001) in methylation levels in tumor relative to normal samples. These levels were determined using probe cg06733329 located 281 bp upstream of the transcription start site of the MXD3 gene. Given that MXD3 splice variants (see next section) share exon 1 these data represent total MXD3 methylation level.

**Table 2:** The MXD3 promoter tends to be hypomethylated in cancer.

**Cancer**	**Δ Methylation beta value (Primary tumor - Solid tissue normal)**	**Sample # (Solid tissue normal)**	**Sample # (Primary tumor)**	***p*-value**	**Statistical significance**
Liver hepatocellular carcinoma	−0.195	50	256	6.20E-36	^***^
Pancreatic adenocarcinoma	−0.115	10	146	1.28E-02	^*^
Breast invasive carcinoma	−0.108	98	743	1.81E-30	^***^
Sarcoma	−0.098	4	242	1.04E-01	ns
Lung squamous cell carcinoma	−0.094	43	361	3.84E-22	^***^
Lung adenocarcinoma	−0.093	32	463	1.54E-12	^***^
Kidney renal clear cell carcinoma	−0.090	160	324	1.85E-42	^***^
Glioblastoma multiforme	−0.088	2	129	6.69E-02	ns
Head & neck squamous cell carcinoma	−0.077	50	528	1.76E-11	^***^
Rectum adenocarcinoma	−0.010	7	98	1.54E-01	ns
Colon adenocarcinoma	0.002	38	302	6.08E-01	ns
Thyroid carcinoma	0.012	56	507	1.34E-01	ns
Prostate adenocarcinoma	0.017	49	340	1.36E-01	ns
Kidney renal papillary cell carcinoma	0.091	45	226	1.34E-11	^***^

Table summary of methylation levels at the MXD3 promoter across TCGA cancer datasets. Shown are the differences in methylation values between primary tumor relative to normal tissue, sample numbers, and statistical test results. Hypomethylated values are highlighted in red and statistical significance was determined by Welch's t-test. Significance,

* p < 0.05,

*** *p < 0.001; ns, not significant*.

### Alternative Splicing of MXD3 Results in two Different Messages and Encoded Proteins

**Figure 1:**
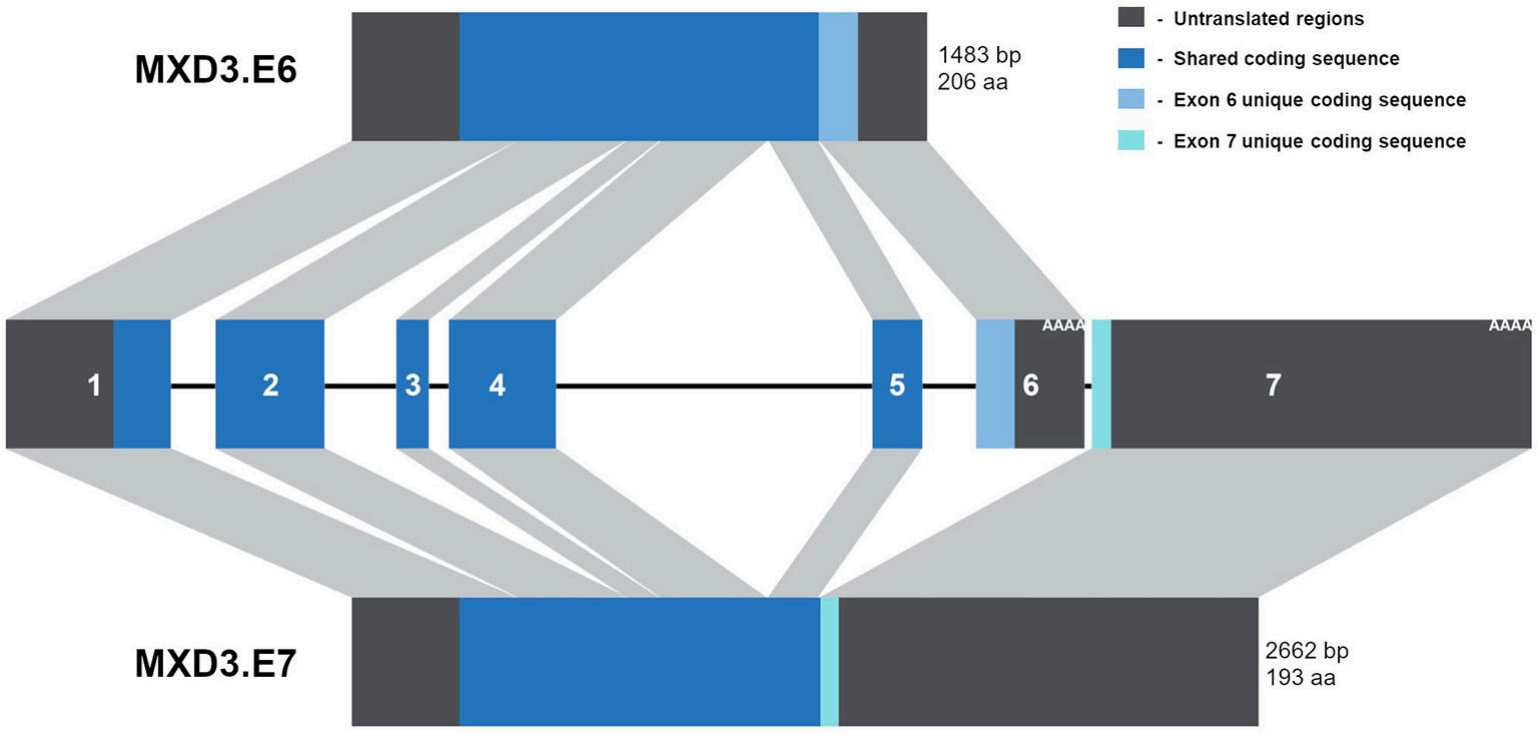
The MXD3 gene contains two alternative polyadenylation signals resulting in two splice variants. Diagram depiction of the MXD3 locus which is made up of 7 exons. These exons are alternatively spliced resulting in MXD3.E6 (exons 1-6) and MXD3.E7 (exons 1-5 and 7). The MXD3.E6 message is shorter with a shorter 3′UTR and encodes a 206 amino acid protein. In contrast, the MXD3.E7 message has a longer 3′UTR; this message results in a shorter protein with a different c-terminus (MXD3.E7 - VLPNENGGTPNHRPTGRGNNISSHH^*^ compared with MXD3.E6 - EELEVDVESLVFGGEAELLRGFVAGQEHSYSHGGGAWL^*^).

From a recent update of NCBI's databases, MXD3 appears to be alternatively spliced resulting in two messages of different lengths. This splice scheme is likely the result of two polyadenylation signals in two different exons (Figure 1). The first splice variant, MXD3.E6, contains exons 1-6 resulting in a 1,483 bp message which encodes a protein of 206 amino acids (Figure 1). The second splice variant, MXD3.E7, contains exons 1-5 but skips exon 6 and terminates with exon 7 resulting in a 2,662 bp message which encodes a protein of 193 amino acids (Figure 1). Based on available cDNA sequence data, both splice variants appear to share the same 5′ untranslated region (UTR) and coding region up to the end of exon 5. Both of the encoded proteins contain the necessary domains for MAX dependent transcription factor functions: Sin3 interacting domain, and the basic, helix-loop-helix, and leucine zipper domain. No known domains could be identified within the c-termini of either MXD3.E6 or MXD3.E7 through online database searches that might underlie differential stability, localization and/or function.

### In Glioblastoma Samples, MXD3.E6 Is the Predominant Form While in Normal Tissue MXD3.E7 Is More Abundant

**Figure 2:**
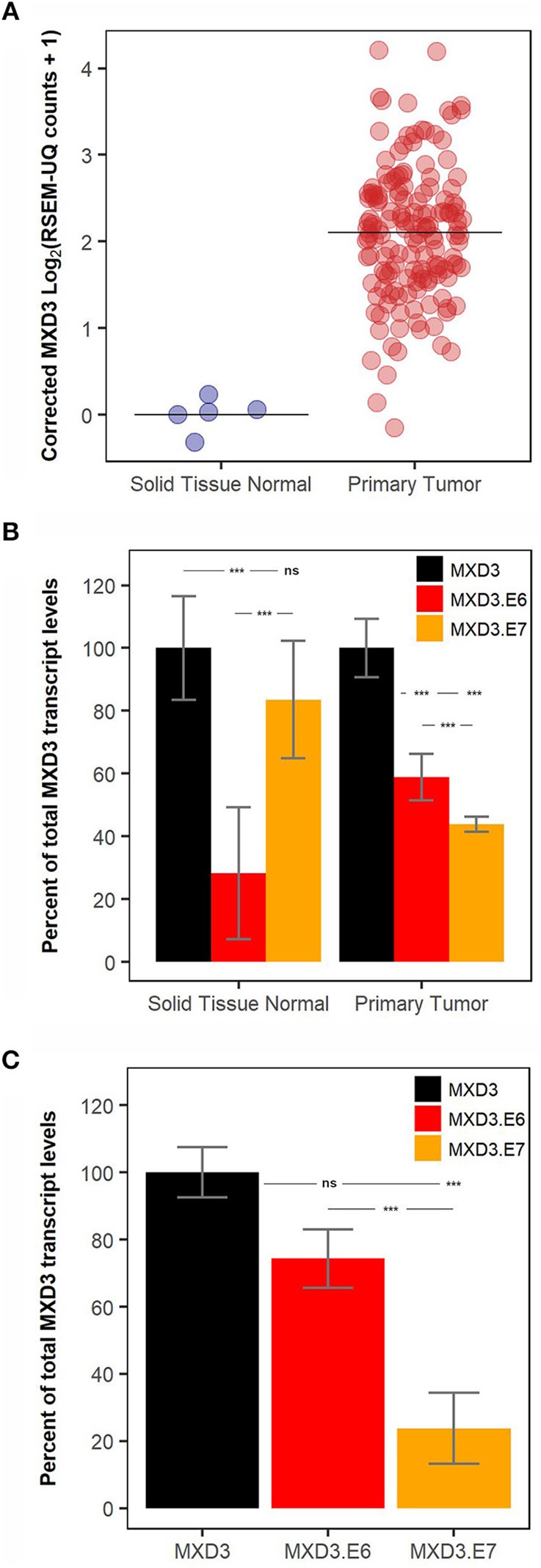
MXD3.E6 is the predominant form in glioblastoma and glioblastoma cell lines whereas MXD3.E7 is the predominant form in normal tissue. **(A)** Total MXD3 transcript levels from TCGA glioblastoma dataset; *N* = 5 and *N* = 154. **(B)** MXD3.E6 and MXD3.E7 transcript levels relative to the measured total MXD3 from TCGA glioblastoma dataset; *N* = 5 and *N* = 154, error bars represent 95% confidence intervals, and statistical significance was determined by one-way ANOVA followed by Tukey's HSD *post hoc* test within each group (normal and primary tumor). **(C)** qPCR measurement using primers specific to MXD3, MXD3.E6, MXD3.E7 transcripts in U87-MG cells undergoing log-phase growth; *N* = 6 across 2 experiments, error bars represent standard error of the mean, and statistical significance was determined by one-way ANOVA followed by Tukey's HSD *post hoc* test. Significance, ^***^p < 0.001; ns, not significant.

Analysis of RNA-seq glioblastoma data available from TCGA reveals that MXD3 transcripts are significantly overexpressed relative to normal tissues (Figure 2A). Within the glioblastoma samples, MXD3.E6 represents 58.87% of the measured total MXD3 population, while MXD3.E7 represents 43.94% of the measured total MXD3 transcript levels (Figure 2B). In the respective normal tissue, MXD3.E7 makes up the majority of the MXD3 transcript population with 83.50% of the measured total MXD3 population (Figures 2A,B). We, additionally, designed qPCR primer pairs probing regions specific to total MXD3, MXD3.E6, and MXD3.E7. We find that in U87-MG cells undergoing log-phase growth, MXD3.E6 makes up the majority of the MXD3 mRNA population, with 74.33% of the measured total MXD3 population (Figure 2C). MXD3.E7 is 23.80% of the measured total MXD3 population and is significantly lower than both total MXD3 and MXD3.E6 (*p* = 7.37E-5 and *p* = 3.45E-3 respectively, one-way ANOVA, Tukey's HSD *post hoc* test).

### The 3′UTR of MXD3.E7 Reduces Protein Expression to a Greater Degree Than MXD3.E6

**Figure 3:**
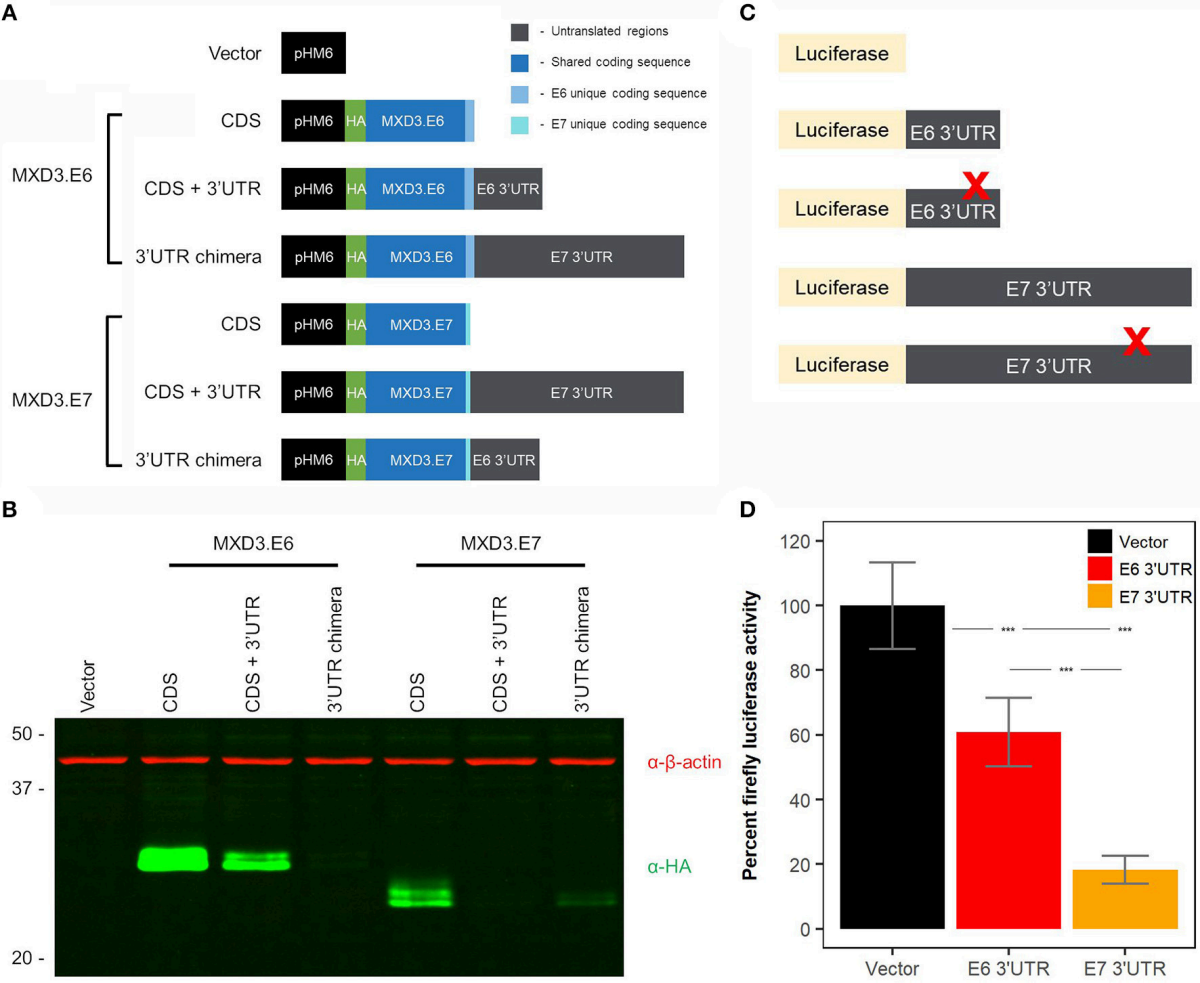
The 3′UTR of MXD3.E7 reduces protein expression to a greater degree than MXD3.E6. **(A)** Exogenous expression of the two splice variants in a glioblastoma cell line. T98G cells were transfected with 2.5 μg of DNA using Lipofectamine 3000. Samples were collected 24 h after transfection, and 15 μg of lysates were run on SDS-PAGE gels. **(B)** Immunoblot against β-actin and HA visualized with LI-COR's Odyssey imaging system. Similar results were obtained in two independent experiments. **(C)** Constructs used in miRNA binding site luciferase screening assays. **(D)** Luciferase activity levels of constructs containing the 3′UTR of MXD3.E6 and E7 fused with firefly luciferase compared to vector control; *N* = 33 across 5 experiments, error bars represent 95% confidence intervals, and statistical significance was determined by one-way ANOVA followed by Tukey's HSD *post hoc* test. Significance, ^***^p < 0.001.

With currently available antibodies we were not able to visualize endogenous MXD3 in either U87-MG or T98G human glioblastoma cell lines (data not shown) although mRNAs are present (Figure 2C). In order to characterize MXD3.E7 we cloned the coding sequence (CDS) into a vector (pHM6) to generate an N-terminally hemagglutinin (HA) tagged construct (Figure 3A). Immunoblot analysis comparing both forms suggests that the two isoforms are expressed at different levels in T98G cells (Figure 3B - compare CDS of MXD3.E6 vs. MXD3.E7).

To analyze the contributions of the 3′UTR of the two splice variants, we generated two additional constructs containing the CDSs of MXD3.E6 and MXD3.E7 and their respective 3′UTRs. Quantitative immunoblotting of MXD3.E6 CDS + 3′UTR signal was reduced by 82.62% compared with the MXD3.E6 CDS alone whereas MXD3.E7 CDS + 3′UTR indicated 98.66% reduced signal compared to its CDS alone counterpart (Figure 3B, fluorescent intensity values in Supplementary Table 1). Signal for a chimeric construct containing the MXD3.E6 CDS with the MXD3.E7 3′UTR (MXD3.E6 3′UTR chimera) was reduced by 99.65% compared with its own 3′UTR, while the signal for chimera of MXD3.E7 CDS with the MXD3.E6 3′UTR (MXD3.E7 3′UTR chimera) was reduced by 90.39% (Figure 3B, fluorescent intensity values in Supplementary Table 1).

Because transfection efficiency differences could underlie the apparent expression level differences between MX3 isoforms, to further validate these results we cloned both 3′UTRs into a firefly luciferase system (Figure 3C). We find that the MXD3.E6 3′UTR significantly reduced luciferase activity by 40.62% (*p* = 2E-7, one-way ANOVA, Tukey's HSD *post hoc* test) and the MXD3.E7 3′UTR significantly reduced luciferase activity by 82.57% (*p* = 0E00, one-way ANOVA, Tukey's HSD *post hoc* test) compared to the vector alone (Figure 3D). The difference in percent luciferase activity between the MXD3.E6 and MXD3.E7 3′UTR is 41.95% (*p* = 1E-07, one-way ANOVA, Tukey's HSD *post hoc* test).

In an attempt to identify possible miRNA binding sites that might explain the differences in expression, we used miRWalk 2.0 to predict miRNA binding sites within the 3′UTRs of both forms of MXD3. We focused on those sites in which their respective miRNA is ectopically expressed in glioblastoma based on available TCGA data. We mutated the seed region of several candidate miRNA binding sites and conducted luciferase assays to determine the effect of these mutations on expression. With the exception of hsa-miR-221 within the 3′UTR of MXD3.E6, all mutants showed no significant differences between their respective controls (Figure 3C, Supplementary Figures 2A,B).

### The Two Isoforms of MXD3 Have Differential Post-translational Modifications

Interestingly, upon longer resolving times and increased resolution through the use of immunoblotting with fluorescently labeled secondary antibodies, compared to previous work (Barisone et al., 2012), we found that both splice variants resolved at two distinct apparent molecular weights (Figure 3B). The additional band suggests the presence of post-translational modification(s). We used several online prediction tools to look at potential phosphorylation, ubiquitination, and SUMOylation sites. We found that both isoforms were highly predicted to be phosphorylated at multiple sites using NetPhos 2.0 (Supplementary Figure 3A). Netphos 2.0 is a machine learning based prediction platform that can be used to identify potential phosphorylation sites (Blom et al., 1999).

**Figure 4:**
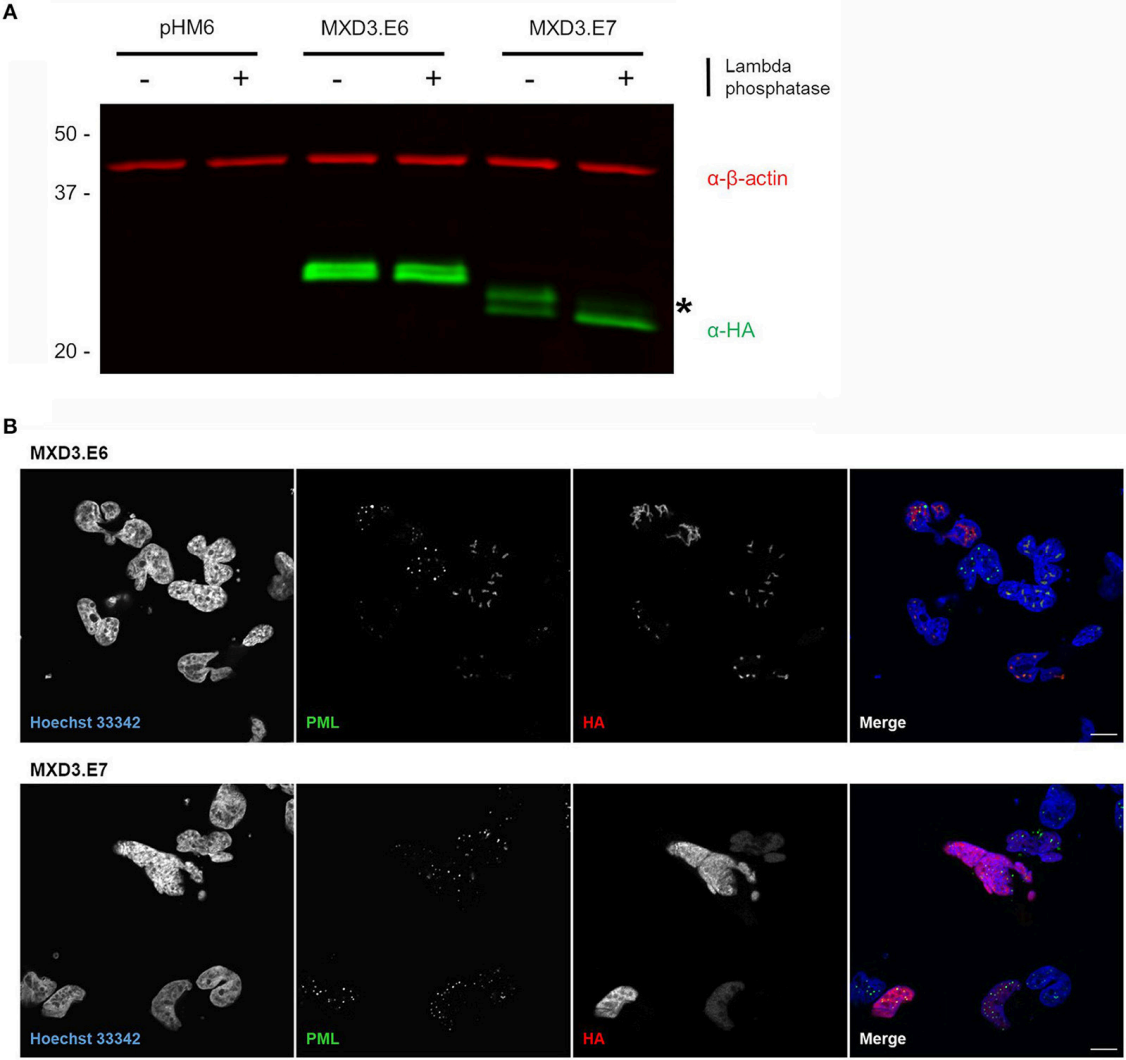
Alternative splicing of MXD3 results in differential post-translational modification and localization of the encoded proteins. **(A)** Immunoblot of transiently expressed HA tagged MXD3.E6 and MXD3.E7 in T98G human glioblastoma cells show that the two splice variants migrate at several distinct apparent molecular weights. There is a band shift upon treatment with phosphatase in MXD3.E7 indicated by asterisk, suggesting that MXD3.E7 is phosphorylated. Similar results were obtained in two independent experiments. **(B)** Immunofluorescence confocal images of transiently expressed HA tagged MXD3.E6 (top) and MXD3.E7 (bottom) in T98G human glioblastoma cells show that the two splice variants are localized to different locales. Specifically, a subset of MXD3.E6 foci are located within subnuclear structures in the vicinity of PML bodies. MXD3.E7, on the other hand, is localized throughout the nucleus. Scale. bar = 10 μm.

We experimentally pursued the possibility of phosphorylation by treating lysates with lambda phosphatase which removes phosphate groups from tyrosine, serine, and threonine (Cohen et al., 1990). Upon treatment, we find that MXD3.E7 shifts to the lower band while MXD3.E6 remain unchanged (Figure 4A). Quantification of the bands reveal that the MXD3.E7 shift is not due to the loss of the top band but rather a shift to the lower band (Figure 4A, fluorescent intensity values in Supplementary Table 2).

Since MXD3.E6 and MXD3.E7 share exons 1-5 (Figure 1) we reasoned that the likely site responsible for the phosphorylation on MXD3.E7 is threonine at position 183 (Supplementary Figure 3A). We mutated threonine 183 to alanine, and subsequently conducted the same phosphatase assay. The results of this assay, however, suggest that threonine 183 is not the site responsible for the modification as MXD3.E7.T183A migrates as a doublet and resolves to a single band upon phosphatase treatment (Supplementary Figure 3B). We then mutated all predicted phosphorylation sites and conducted a screen to identify the responsible site (Supplementary Figure 3C). None of the single mutants resulted in the disappearance of the heavier, presumably, phosphorylated form of MXD3.E7 (Supplementary Figure 3C).

### The Two Splice Variants Localize to Different Nuclear Locales

Immunocytochemistry visualization of both splice variants reveal that the two splice variants localize to different nuclear locales. MXD3.E7 is found throughout the nucleus whereas MXD3.E6 is predominantly found as nuclear foci-like structures in T98G cells (Figure 4B) and in U87-MG cells (Supplementary Figure 4). In T98G cells some MXD3.E6 foci are located in the vicinity of subnuclear structures marked by immunostaining with the promyelocytic leukemia (PML) protein (Figure 4B).

## Discussion

Datasets from TCGA have been invaluable for exploratory analyses within the context of cancers and our analysis has provided useful insights into MXD3 expression in cancer. First, we found that MXD3 message levels are significantly higher in cancers relative to their normal tissue counterparts, specifically in 13/18 (~72%) of the datasets examined. This result could be interpreted in multiple ways: copy number changes to the MXD3 gene, misregulation of upstream MXD3 transcriptional regulator, and/or misregulation of a regulator of the MXD3 message. The first possibility can be eliminated since we find that the MXD3 gene is infrequently altered; in terms of changes in sequence and copy number. For the second possibility, we find that the upstream promoter region to the MXD3 appears to be hypomethylated, suggesting the possibility of a misregulation of upstream regulator of MXD3 transcription. E2F1, for example, is known to have a binding site upstream of the MXD3 gene (Fox and Wright, 2003). This site is important for the S-phase specific expression of MXD3 within cycling cells (Fox and Wright, 2001). Such upstream regulators may be important for the overexpression of MXD3 in human cancers. Future work is necessary to determine which upstream regulator(s) might be involved.

We explored the possibility of misregulation of the MXD3 message by examining alternative splicing of MXD3. Specifically, APA of the MXD3 transcript allows for two distinct transcripts: MXD3.E6 and MXD3.E7 (Figure 1). Furthermore, the splicing results in two different CDS with the protein encoded by MXD3.E7 being slightly smaller (Figure 1). Currently, there is no cDNA evidence for the presence of MXD3.E7 in non-human cells; which could be because it is not expressed at high enough levels to be identified in cDNA libraries. There, however, does appear to be experimental evidence for a longer transcript of similar size differences in mouse cells (Quéva et al., 1998). We focused our efforts in GBM, which showed the highest fold overexpression from the TCGA datasets examined. GBM is one of the most common and aggressive primary brain tumors (Odjélé et al., 2012). It largely affects adults, with 95% of cases occurring in adults, and 5% in children (Odjélé et al., 2012). Many of the drivers directly responsible for this disease have been identified, including EGFR, RAS, PI3K/PTEN/AKT, RB, and the TP53 pathway (Crespo et al., 2015). The EGFR gene, for example, is frequently amplified leading to its overexpression in GBM (Crespo et al., 2015). This overexpression results in increased proliferation and tumor survival (Crespo et al., 2015). In contrast, RAS signaling is overactive despite its genomic locus being infrequently altered in GBM (Crespo et al., 2015). These drivers have been well characterized through several studies including multi-dimensional sequencing studies in GBM (Network, 2013). Due to the complexity of GBM, however, there remains the possibility of previously unknown contributors to this disease such as MXD3.

Based on available TCGA data, we find that the MXD3.E7 is expressed at higher levels in normal cells when compared to cancer cells (Figure 2B). On the other hand, MXD3.E6 mRNA is expressed at higher levels in cancer cells compared to normal cells (Figure 2B). Furthermore, U87-MG cells predominantly express MXD3.E6 transcripts (Figure 2C). These data support a model in which expression of MXD3.E6, which is 1179 bp shorter than MXD3.E7, allows for cancer cells to escape miRNA regulation of the transcript (Sandberg et al., 2008; Mayr and Bartel, 2009). When we experimentally examined the 3′UTR of the two splice variants of MXD3, we find that MXD3.E7, which contains a much longer 3′UTR, results in a larger reduction in expression compared to MXD3.E6 (Figure 3).

The mechanism behind the differential contributions of the 3′UTRs may be due to RNA secondary structure stabilization, RNA binding destabilization elements, and/or miRNA regulation of the transcript. The experimental validation of predicted miRNA binding sites on the 3′UTR is the focus of our current efforts. As a preliminary screen we produced 12 3′UTR mutants of MXD3.E6 and 11 3′UTR mutants of MXD3.E7. Preliminary luciferase assays with their respective controls did not reveal any significant differences, with the exception of hsa-miR-221 for MXD3.E6, we found no significant difference (Supplementary Figures 2A,B). These efforts were an initial attempt to narrow down the potential miRNA binding sites responsible for the differential expression between MXD3.E6 and MXD3.E7. There still remains, however, the possibilities that secondary RNA structures, and/or destabilization elements such as binding to the 3′UTRs can lead to stabilization or destabilization of mRNA sequences (Garneau et al., 2007; Tian and Manley, 2017). Future efforts to narrow down the primary contributor to the differential expression would help in our understanding of how APA of MXD3 regulates its levels and possibly for other genes that undergo APA.

Since the two splice variants have different CDS and 3′UTRs, we also examined functional differences between the two encoded proteins. In our previous studies in other cancer cell lines, knockdown and overexpression of MXD3 leads to reduced cell numbers (Yun et al., 2007; Barisone et al., 2012, 2015; Ngo et al., 2014). We cannot confirm knock-down of endogenous MXD3 protein because existing anti-MXD3 antibodies do not recognize endogenous protein in our hands. In preliminary overexpression studies, we observed a small (approximately 20%) decrease in T98G proliferation for MXD3.E6 but not for MXD3.E7; however, this effect could be due to the apparent expression level differences observed between the isoforms (Figure 3B) and requires further validation. For these reasons, we focused the rest of our subsequent efforts on characterizing the modification and localization differences between the splice variants via exogenous expression. To that end, MXD3.E7 appears to be phosphorylated whereas MXD3.E6 is not (Figure 4A). This phosphorylation appears to be due to more than one site on the protein as mutation of individual amino acid residues do not alter mobility (Supplementary Figure 3A). Phosphorylation of MXD3.E7 may be part of a mechanism in which the cell downregulates its levels. Experimental determination of the exact site of phosphorylation and the use of subsequent mutants will be crucial in further elucidating this mechanism.

We also find that the two isoforms localize to different nuclear structures. MXD3.E7 localizes throughout the nucleus whereas MXD3.E6 localizes predominantly to nuclear foci (Figure 4B). Some MXD3.E6 nuclear foci are located near areas marked by immunostaining with the promyelocytic leukemia protein (PML), a marker of PML bodies (Figure 4B). PML bodies are nuclear structures that are thought to be the site for recruitment of nuclear proteins in order to enact post-translational modification on them (Lallemand-Breitenbach and de The, 2010; Guan and Kao, 2015). MXD3.E6 does appear to be modified due to the presence of the protein migrating at two apparent molecular weights (Figure 4A). The type of modification that MXD3.E6 undergoes does not appear to be phosphorylation (Figure 4A). Determination of the type of post-translational modification would be helpful in determining the significance of the subset of MXD3.E6 located nearby PML bodies in T98G cells. One such modification is ubiquitination, the possibility of which we are actively pursuing. Altogether, alternative splicing of MXD3 allows for differential nuclear localization and post-translational modification of the encoded MXD3 protein.

Based on currently available cDNA data it appears that MXD3 is the only member of the MYC/MAX/MXD transcriptional network to undergo APA. An interesting possibility is that MXD3 may belong to another non-canonical (MYC/MAX/MXD) group of APA genes. This subgroup of genes can be quickly up/down regulated via splicing one message or the other. In the context of MXD3 and the MYC/MAX/MXD network the adjustment may have profound cellular effects. MXD3 levels have been shown to be important for cell proliferation. A current proposed model of how this occurs within the context of the MYC/MAX/MXD transcriptional network is that MXD and MYC family members compete for MAX binding in order to enact different functional outcomes; for example, the balance between cell proliferation and differentiation. With MXD3 being the only member of the network to undergo APA, this may be a separate pathway to adjust only MXD3 levels which in effectively changes the balance between MXD and MYC levels.

In summary, we demonstrate that MXD3 has two splice forms likely resulting from APA that are differentially expressed in cancer and normal tissue. The proteins encoded by the splice forms are different molecular weights, undergo different post-translation modification and localize to different areas of the nucleus. Future work is needed to determine the molecular mechanisms that underlie the differences between the splice forms and their roles in cell proliferation.

## Author Contributions

TN and ED conceived and designed experiments. TN, AC, TB, and SE performed experiments. TN, AC, TB, and SE analyzed data. TN, KSL, and ED wrote and critically edited the manuscript.

### Conflict of Interest Statement

The authors declare that the research was conducted in the absence of any commercial or financial relationships that could be construed as a potential conflict of interest.

## References

[B1] Ayer D. E. Kretzner L. Eisenman R. N. (1993). Mad: a heterodimeric partner for Max that antagonizes Myc transcriptional activity. Cell 72, 211–222. 10.1016/0092-8674(93)90661-9 8425218

[B2] Ayer D. E. Laherty C. D. Lawrence Q. A. Armstrong A. P. Eisenman R. N. (1996). Mad proteins contain a dominant transcription repression domain. Mol. Cell Biol. 16, 5772–5781. 10.1128/MCB.16.10.5772 8816491 PMC231578

[B3] Barisone G. A. Ngo T. Tran M. Cortes D. Shahi M. H. Nguyen T. V.. (2012). Role of MXD3 in proliferation of DAOY human medulloblastoma cells. PLoS ONE 7:e38508. 10.1371/journal.pone.0038508 22808009 PMC3393725

[B4] Barisone G. A. Satake N. Lewis C. Duong C. Chen C. Lam K. S.. (2015). Loss of MXD3 induces apoptosis of Reh human precursor B acute lymphoblastic leukemia cells. Blood Cells Mol. Dis. 54, 329–335. 10.1016/j.bcmd.2014.12.002 25554682 PMC4387021

[B5] Barisone G. A. Yun J. S. Diaz E. (2008). From cerebellar proliferation to tumorigenesis: new insights into the role of Mad3. Cell Cycle 7, 423–427. 10.4161/cc.7.4.5413 18235219

[B6] Blom N. Gammeltoft S. Brunak S. (1999). Sequence and structure-based prediction of eukaryotic protein phosphorylation sites. J. Mol. Biol. 294, 1351–1362. 10.1006/jmbi.1999.3310 10600390

[B7] Cerami E. Gao J. Dogrusoz U. Gross B. E. Sumer S. O. Aksoy B. A.. (2012). The cBio cancer genomics portal: an open platform for exploring multidimensional cancer genomics data. Cancer Discov. 2, 401–404. 10.1158/2159-8290.CD-12-0095 22588877 PMC3956037

[B8] Cohen P. T. Brewis N. D. Hughes V. Mann D. J. (1990). Protein serine/threonine phosphatases; an expanding family. FEBS Lett. 268, 355–359. 10.1016/0014-5793(90)81285-V 2166691

[B9] Crespo I. Vital A. L. Gonzalez-Tablas M. Patino Mdel C. Otero A. Lopes M. C.. (2015). Molecular and genomic alterations in glioblastoma multiforme. Am. J. Pathol. 185, 1820–1833. 10.1016/j.ajpath.2015.02.023 25976245

[B10] Di Giammartino D. C. Nishida K. Manley J. L. (2011). Mechanisms and consequences of alternative polyadenylation. Mol. Cell. 43, 853–866. 10.1016/j.molcel.2011.08.017 21925375 PMC3194005

[B11] Díez-Villanueva A. Mallona I. Peinado M. A. (2015). Wanderer, an interactive viewer to explore DNA methylation and gene expression data in human cancer. Epigenetics Chromatin 8:22. 10.1186/s13072-015-0014-8 26113876 PMC4480445

[B12] Dweep H. Gretz N. (2015). miRWalk2.0: a comprehensive atlas of microRNA-target interactions. Nat. Methods 12:697. 10.1038/nmeth.3485 26226356

[B13] Fox E. J. Wright S. C. (2001). S-phase-specific expression of the Mad3 gene in proliferating and differentiating cells. Biochem. J. 359(Pt 2), 361–367. 10.1042/bj3590361 11583582 PMC1222154

[B14] Fox E. J. Wright S. C. (2003). The transcriptional repressor gene Mad3 is a novel target for regulation by E2F1. Biochem. J. 370(Pt 1), 307–313. 10.1042/bj20021583 12444919 PMC1223166

[B15] Gao J. Aksoy B. A. Dogrusoz U. Dresdner G. Gross B. Sumer S. O.. (2013). Integrative analysis of complex cancer genomics and clinical profiles using the cBioPortal. Sci. Signal 6:pl1. 10.1126/scisignal.2004088 23550210 PMC4160307

[B16] Garneau N. L. Wilusz J. Wilusz C. J. (2007). The highways and byways of mRNA decay. Nat. Rev. Mol. Cell Biol. 8, 113–126. 10.1038/nrm2104 17245413

[B17] Goldman M. Craft B. Swatloski T. Ellrott K. Cline M. Diekhans M.. (2013). The UCSC cancer genomics browser: update 2013. Nucleic Acids Res. 41, D949–D954. 10.1093/nar/gks1008 23109555 PMC3531186

[B18] Grandori C. Cowley S. M. James L. P. Eisenman R. N. (2000). The Myc/Max/Mad network and the transcriptional control of cell behavior. Annu. Rev. Cell. Dev. Biol. 16, 653–699. 10.1146/annurev.cellbio.16.1.653 11031250

[B19] Guan D. Kao H. Y. (2015). The function, regulation and therapeutic implications of the tumor suppressor protein, PML. Cell Biosci. 5:60. 10.1186/s13578-015-0051-9 26539288 PMC4632682

[B20] Hurlin P. J. Queva C. Koskinen P. J. Steingrimsson E. Ayer D. E. Copeland N. G.. (1995). Mad3 and Mad4: novel Max-interacting transcriptional repressors that suppress c-myc dependent transformation and are expressed during neural and epidermal differentiation. EMBO J. 14, 5646–5659. 10.1002/j.1460-2075.1995.tb00252.x 8521822 PMC394680

[B21] Kretzner L. Blackwood E. M. Eisenman R. N. (1992). Myc and Max proteins possess distinct transcriptional activities. Nature 359, 426–429. 10.1038/359426a0 1406956

[B22] Lallemand-Breitenbach V. de The H. (2010). PML nuclear bodies. Cold Spring Harb. Perspect. Biol. 2:a000661. 10.1101/cshperspect.a000661 20452955 PMC2857171

[B23] Mayr C. Bartel D. P. (2009). Widespread shortening of 3'UTRs by alternative cleavage and polyadenylation activates oncogenes in cancer cells. Cell 138, 673–684. 10.1016/j.cell.2009.06.016 19703394 PMC2819821

[B24] Network T. C. (2013). Corrigendum: comprehensive genomic characterization defines human glioblastoma genes and core pathways. Nature 494:506. 10.1038/nature11903 23389443

[B25] Ngo T. Barisone G. A. Lam K. S. Diotaaz E. (2014). MXD3 regulation of DAOY cell proliferation dictated by time course of activation. BMC Cell Biol. 15:30. 10.1186/1471-2121-15-30 25053245 PMC4226952

[B26] Odjélé A. Charest D. Morin P. Jr. (2012). miRNAs as important drivers of glioblastomas: a no-brainer? Cancer Biomark 11, 245–252. 10.3233/CBM-2012-0271 23248182 PMC13016227

[B27] Quéva C. Hurlin P. J. Foley K. P. Eisenman R. N. (1998). Sequential expression of the MAD family of transcriptional repressors during differentiation and development. Oncogene 16, 967–977. 10.1038/sj.onc.1201611 9519870

[B28] Quéva C. McArthur G. A. Iritani B. M. Eisenman R. N. (2001). Targeted deletion of the S-phase-specific Myc antagonist Mad3 sensitizes neuronal and lymphoid cells to radiation-induced apoptosis. Mol. Cell Biol. 21, 703–712. 10.1128/M.C.B.21.3.703-712.2001 11154258 PMC86662

[B29] R Core Team (2017). R: A Language and Environment for Statistical Computing. Vienna: R Foundation for Statistical Computing.

[B30] Sanborn J. Z. Benz S. C. Craft B. Szeto C. Kober K. M. Meyer L.. (2011). The UCSC cancer genomics browser: update 2011. Nucleic Acids Res. 39, D951–D959. 10.1093/nar/gkq1113 21059681 PMC3013705

[B31] Sandberg R. Neilson J. R. Sarma A. Sharp P. A. Burge C. B. (2008). Proliferating cells express mRNAs with shortened 3' untranslated regions and fewer microRNA target sites. Science 320, 1643–1647. 10.1126/science.1155390 18566288 PMC2587246

[B32] Shi Y. (2012). Alternative polyadenylation: new insights from global analyses. RNA 18, 2105–2117. 10.1261/rna.035899.112 23097429 PMC3504663

[B33] Takagaki Y. Seipelt R. L. Peterson M. L. Manley J. L. (1996). The polyadenylation factor CstF-64 regulates alternative processing of IgM heavy chain pre-mRNA during B cell differentiation. Cell 87, 941–952. 10.1016/S0092-8674(00)82000-0 8945520

[B34] Tian B. Manley J. L. (2017). Alternative polyadenylation of mRNA precursors. Nat. Rev. Mol. Cell Biol. 18, 18–30. 10.1038/nrm.2016.116 27677860 PMC5483950

[B35] Velculescu V. E. Zhang L. Vogelstein B. Kinzler K. W. (1995). Serial analysis of gene expression. Science 270, 484–487. 10.1126/science.270.5235.484 7570003

[B36] Yun J. S. Rust J. M. Ishimaru T. Diaz E. (2007). A novel role of the Mad family member Mad3 in cerebellar granule neuron precursor proliferation. Mol. Cell Biol. 27, 8178–8189. 10.1128/MCB.00656-06 17893326 PMC2169189

[B37] Zhu J. Sanborn J. Z. Benz S. Szeto C. Hsu F. Kuhn R. M.. (2009). The UCSC cancer genomics browser. Nat Methods 6, 239–240. 10.1038/nmeth0409-239 19333237 PMC5027375

