# Supplementary material for: Alternative Splicing of MXD3 and Its Regulation of MXD3 Levels in Glioblastoma
Source: Front Mol Biosci. 2019 Feb 19;6:5. doi: 10.3389/fmolb.2019.00005 (PMC6390498; doi:10.3389/fmolb.2019.00005)

Supplementary Information

1. Supplementary Figures 1-4
2. Supplementary Tables 1-2
3. Uncropped immunoblots

1. Supplementary Figures 1-4

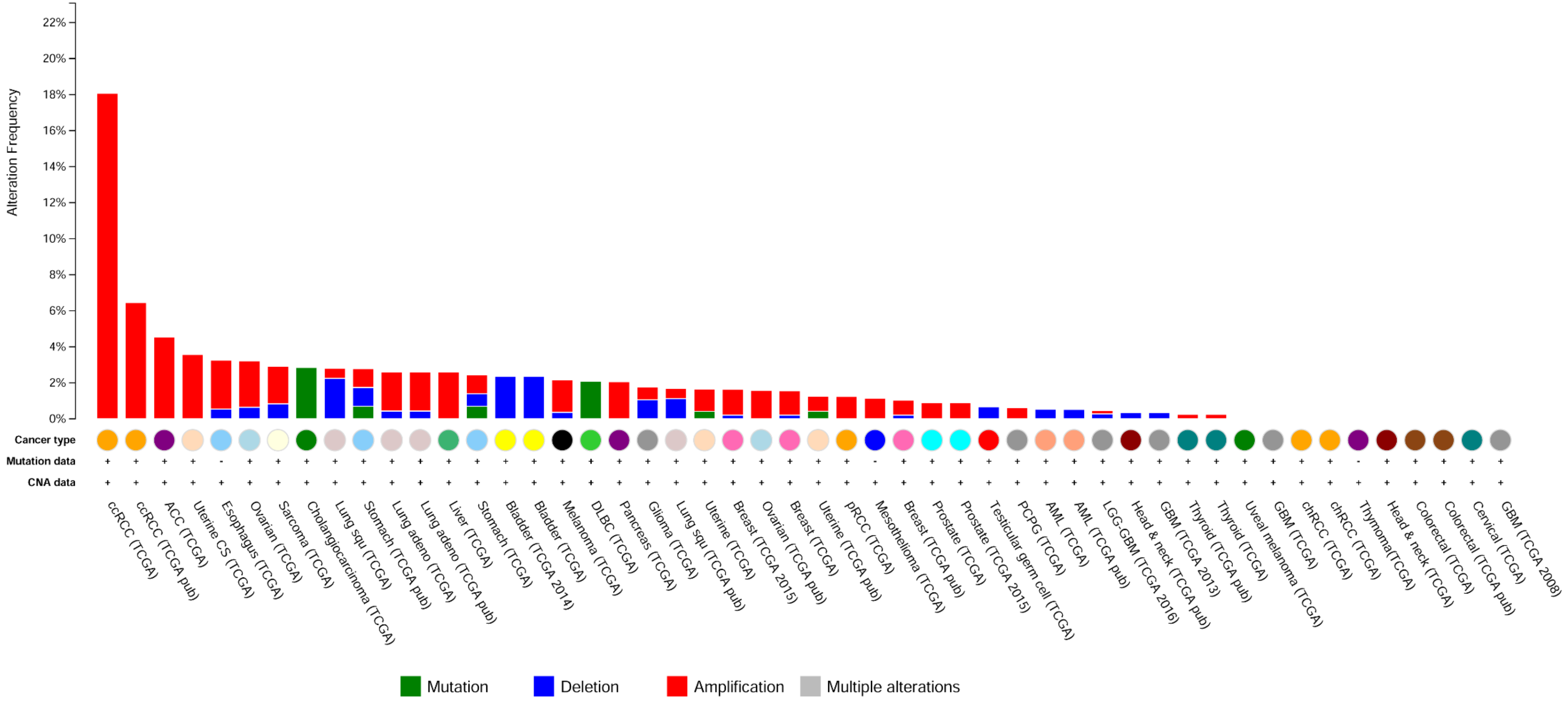

Supplementary Figure 1 - MXD3 gene alteration frequencies are low across TCGA cancer datasets. MXD3 alteration frequencies across TCGA cancer datasets visualized using cBioPortal for Cancer Genomics' platform. Shown in green are those samples which have the MXD3 gene has been mutated, blue are samples in which the MXD3 gene is deleted, in red are those samples in which the MXD3 gene is amplified, and lastly in grey are those in which there are multiple alterations to the MXD3 gene.

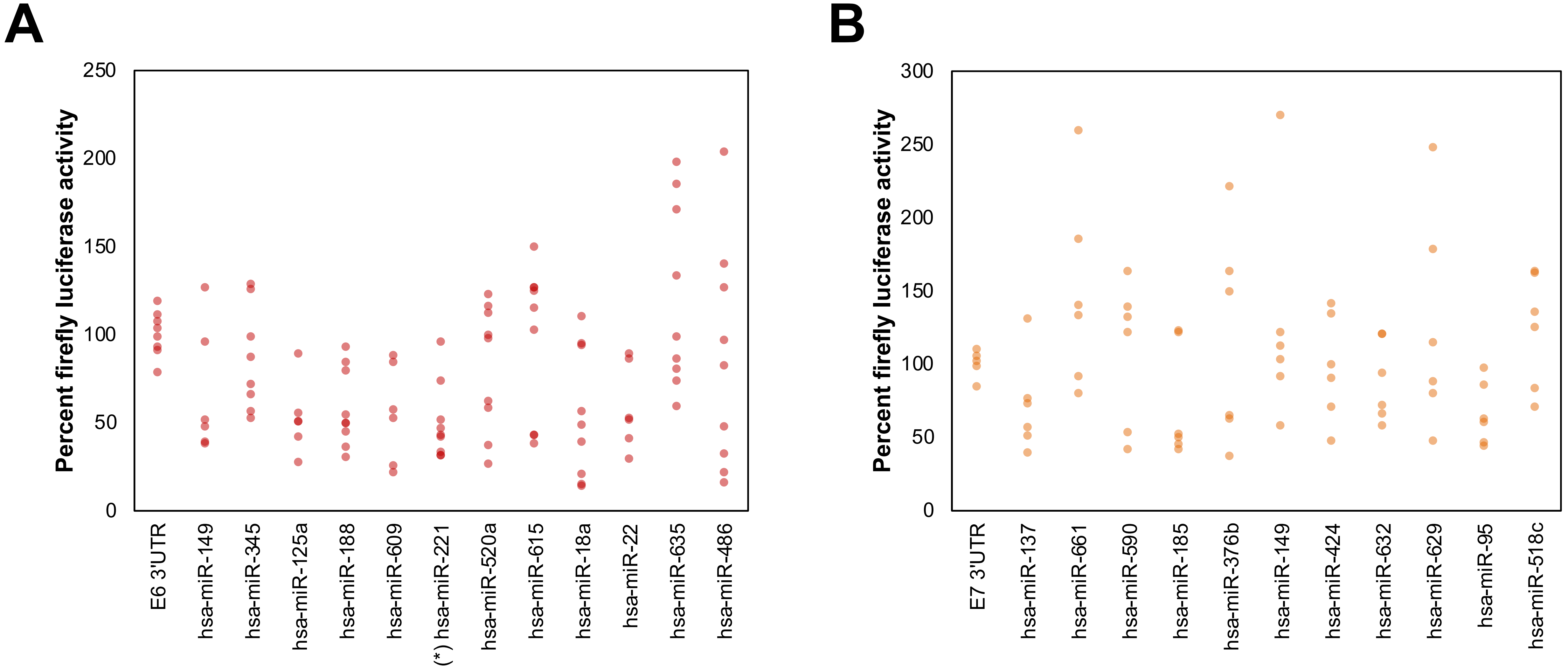

Supplementary Figure 2 – Screen of potential miRNA binding sites in the 3'UTRs of MXD3.E6 and MXD3.E7
Luciferase assays with mutant constructs of predicted miRNA binding sites within the 3'UTRs of MXD3.E6 (**A**) and MXD3.E7 (**B**). With the exception of has-miR-221 (denoted with an asterisk in **A**), no significant difference was detected between mutants relative to control.

A

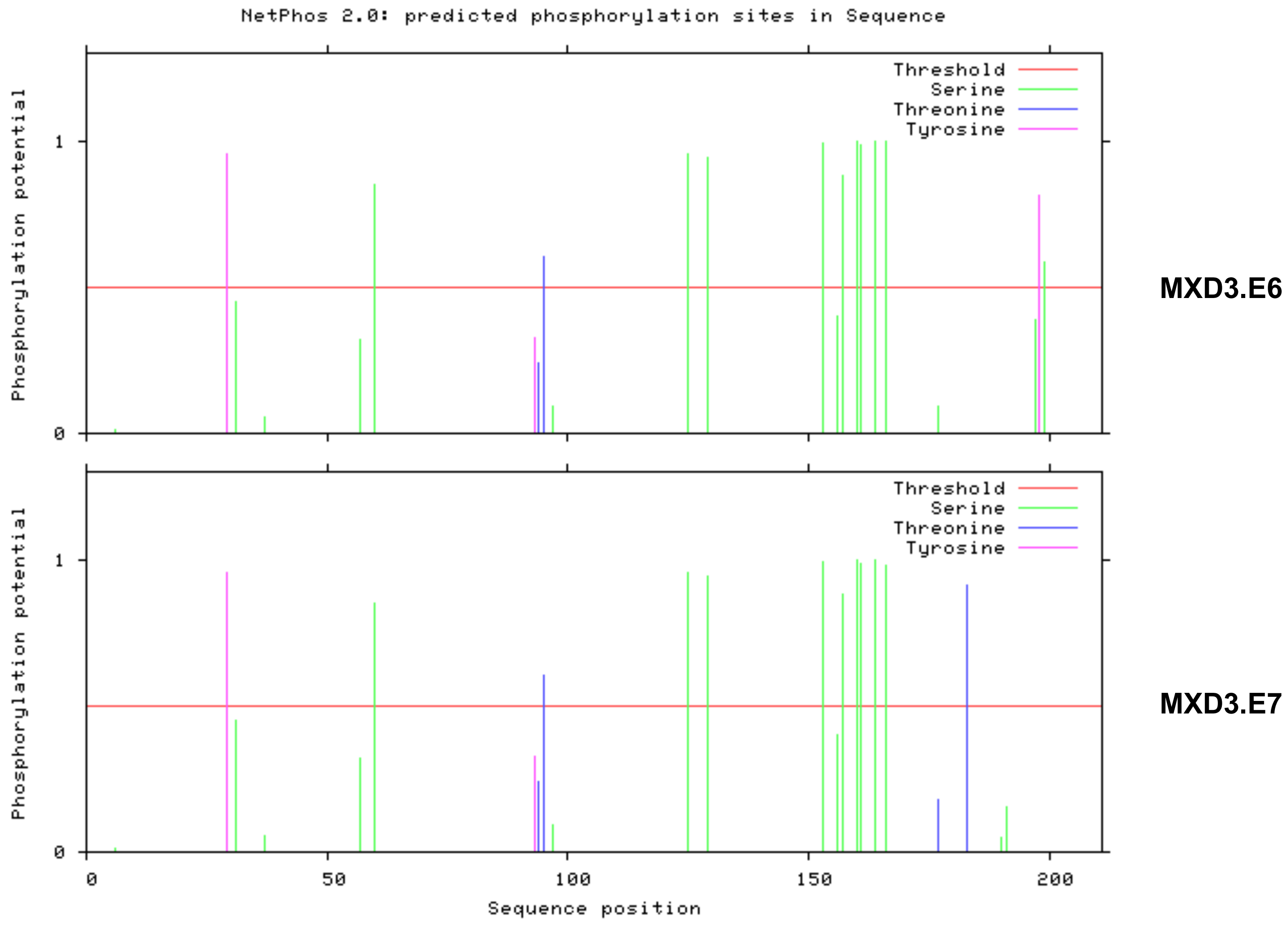

Supplementary Figure 3 - Post-translational modifications of MXD3.E6 and MXD3.E7

(A) NetPhos 2.0 analysis of amino acids in MXD3 predict that threonine 183 in MXD3.E7 is the most likely site of phosphorylation due to it being the highest predicted unique phosphorylation site on MXD3.E7 compared to MXD3.E6.

B

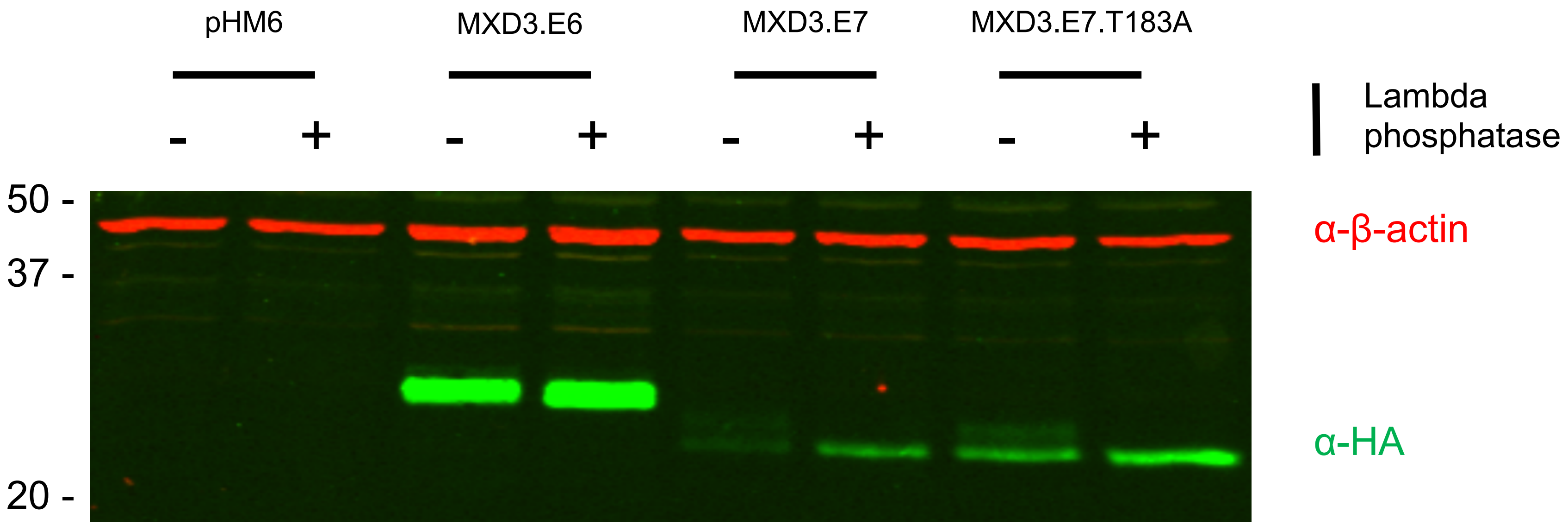

Supplementary Figure 3 - Post-translational modifications of MXD3.E6 and MXD3.E7
(**B**) MXD3E7.T183A is still able to be phosphorylated. Note the upper band in both MXD3.E7 (-) and MXD3.E7.T183A (-) lanes that is absent upon lambda phosphatase treatment (+).

C

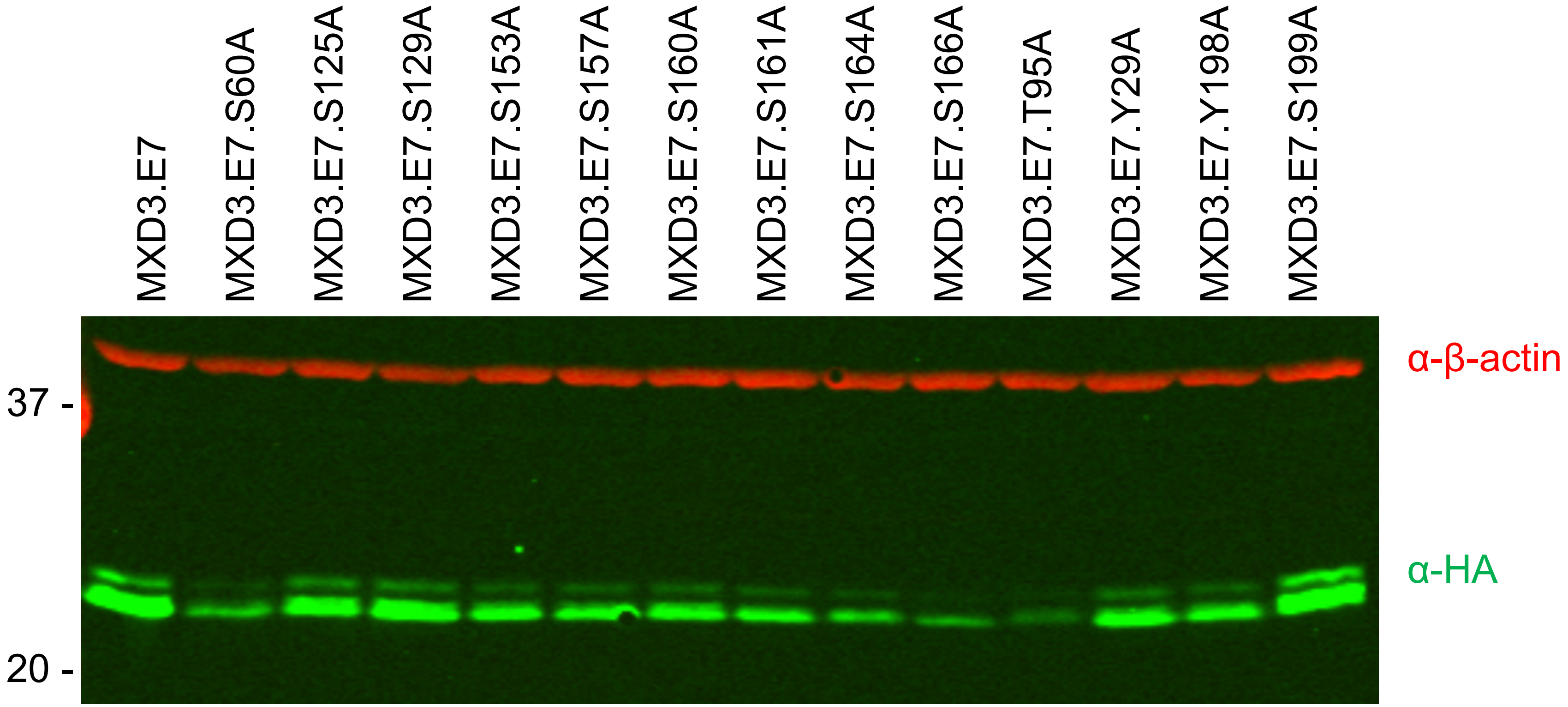

Supplementary Figure 3 - Post-translational modifications of MXD3.E6 and MXD3.E7
(**C, D**) All other single mutants appear to be phosphorylated. Note the presence of the upper band in all lanes.

D

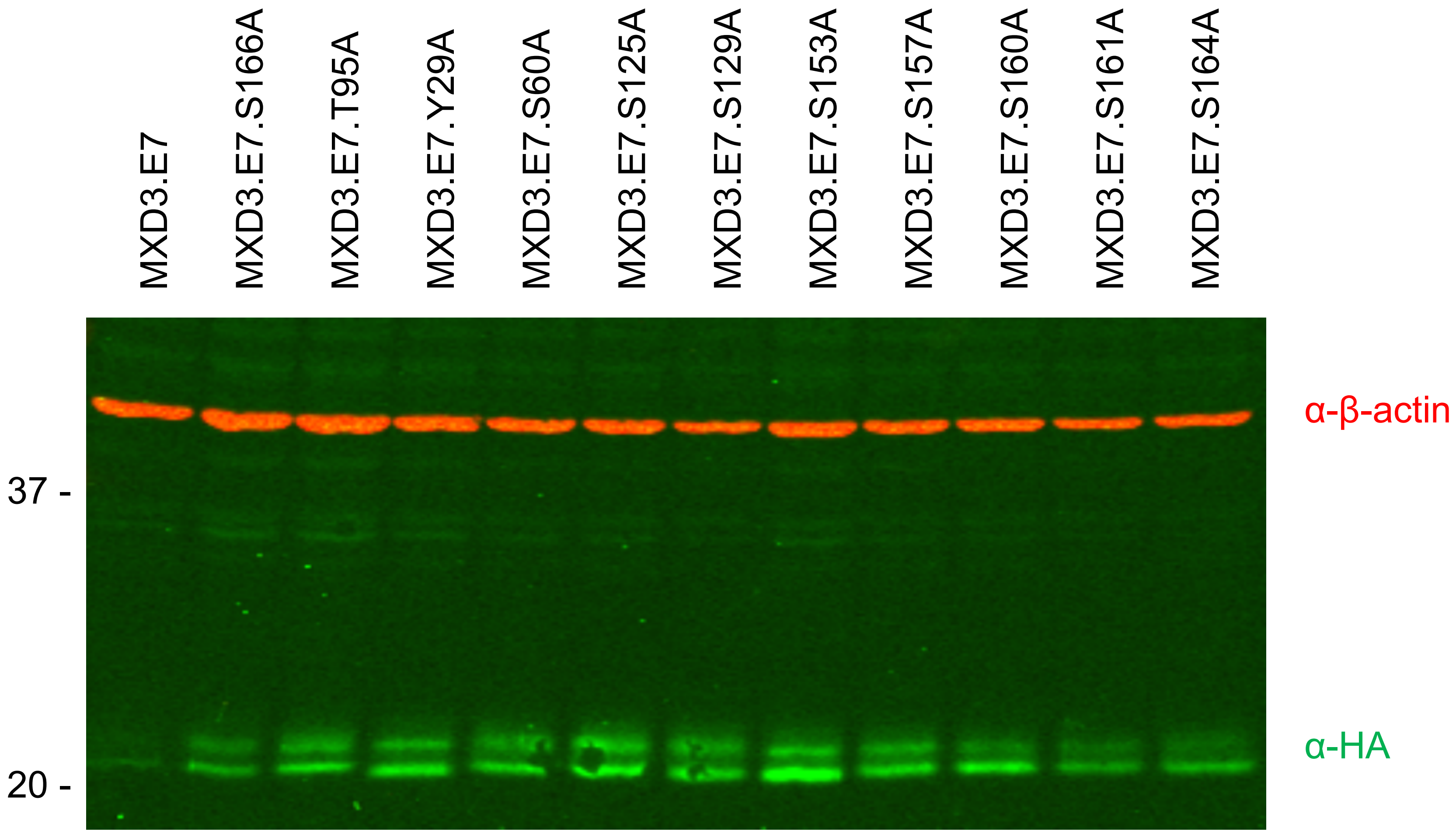

Supplementary Figure 3 - Post-translational modifications of MXD3.E6 and MXD3.E7
(**C, D**) All other single mutants appear to be phosphorylated. Note the presence of the upper band in all lanes.

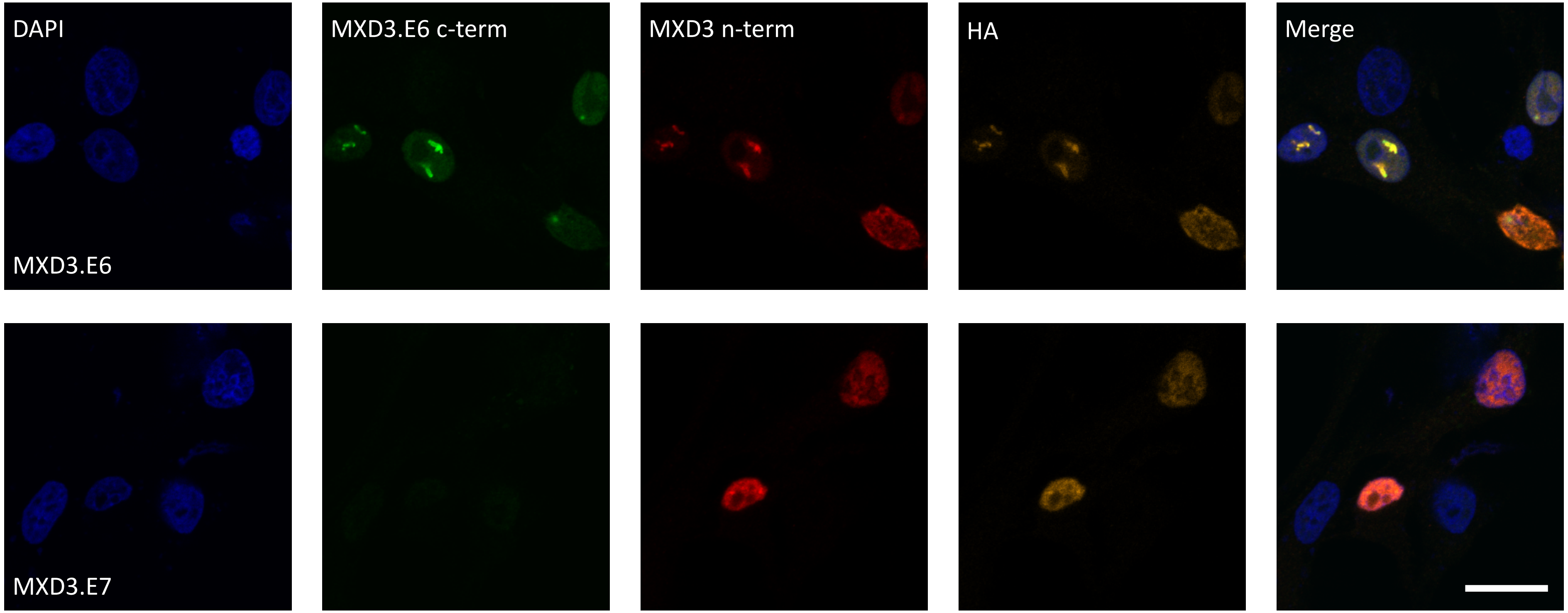

Supplementary Figure 4 - Post-translational modifications of MXD3.E6 and MXD3.E7
Immunofluorescence confocal images of transiently expressed HA tagged MXD3.E6 (top) and MXD3.E7 (bottom) in U87 human glioblastoma cells show that the two splice variants are localized to different locales. MXD3 n-term (RAB-anti-MXD3 - AbCam - ab50729) antibody targets the n-terminus of MXD3, which both forms share in sequence. MXD3.E6 c-term (MUS-anti MXD3 - Neuromab - 75-250) targets the c-terminal sequence of MXD3.E6. Scale bar = 10 µm.

2. Supplementary Tables 1-2

Supplementary Table 1 – Quantification for data shown in Figure 3B

Lane	Sample	Form	Normalized signal	Relative normalized signal
1	Vector		2112.857143	
2	CDS	MXD3.E6	5502995.169	100
3	CDS + 3'UTR	MXD3.E6	956151.6793	17.37511391
4	3'UTR chimera	MXD3.E6	19035.89744	0.345918847
5	CDS	MXD3.E7	703674.3003	100
6	CDS + 3'UTR	MXD3.E7	9370	1.331581954
7	3'UTR chimera	MXD3.E7	67600.47226	9.606784308

Supplementary Table 2 – Quantification for data shown in Figure 4A

Form	Treatment	Band	Normalized signal	Relative normalized signal
MXD3.E6	Control	Top	730038	0.51
		Bottom	691615	0.49
		Both	1422692	
	Lambda phosphatase	Top	539000	0.46
		Bottom	655000	0.56
		Both	1180000	
MXD3.E7	Control	Top	373371	0.56
		Bottom	259200	0.39
		Both	665486	
	Lambda phosphatase	Top	146498	0.20
		Bottom	549653	0.74
		Both	747256	

3. Uncropped immunoblots

Figure 3B

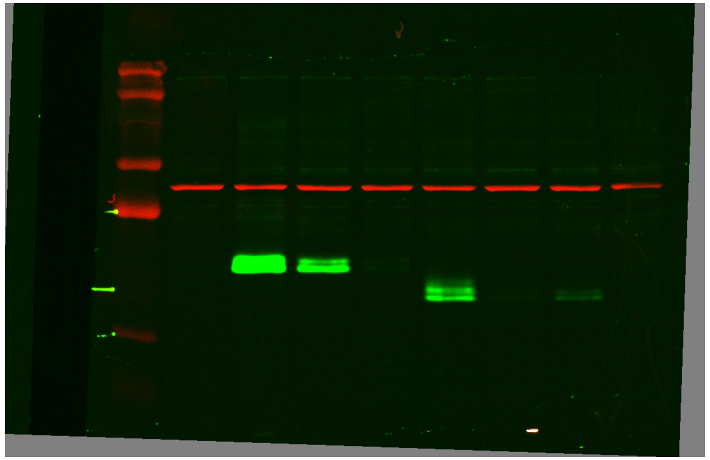

Figure 4A

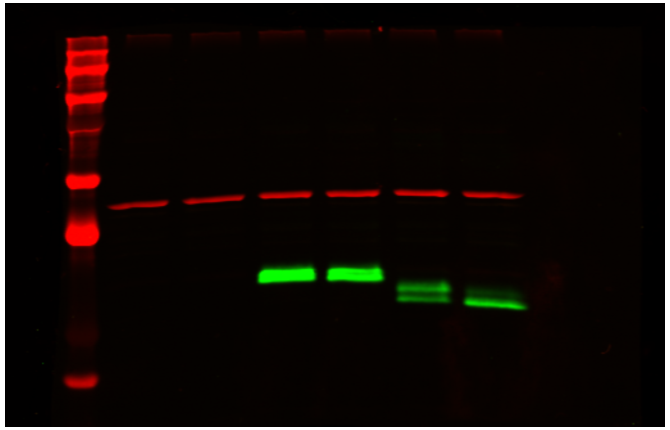

Supplementary Figure 2B

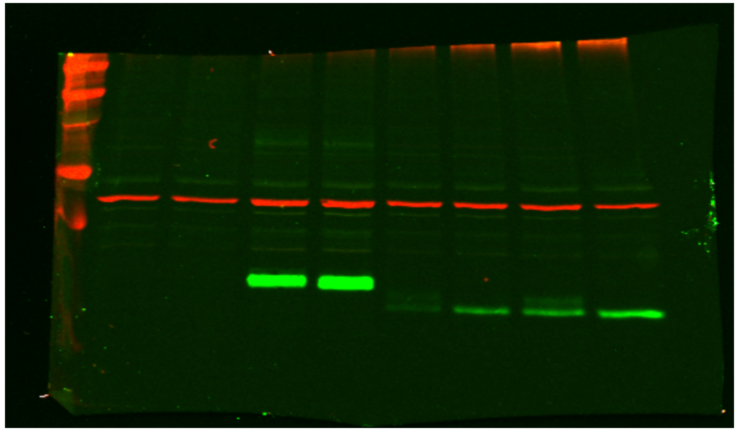

Supplementary Figure 2C

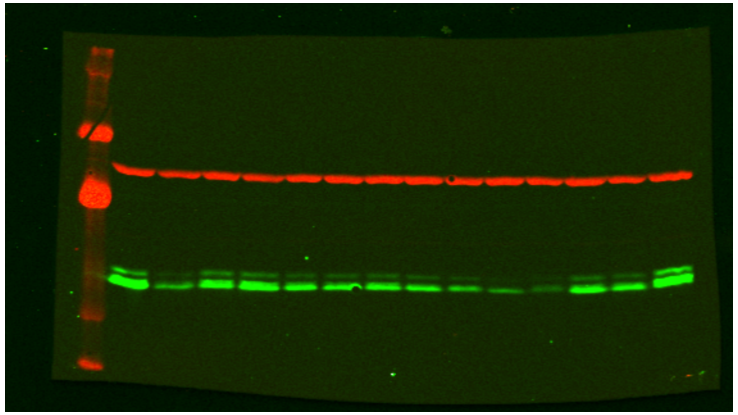

Supplementary Figure 2D

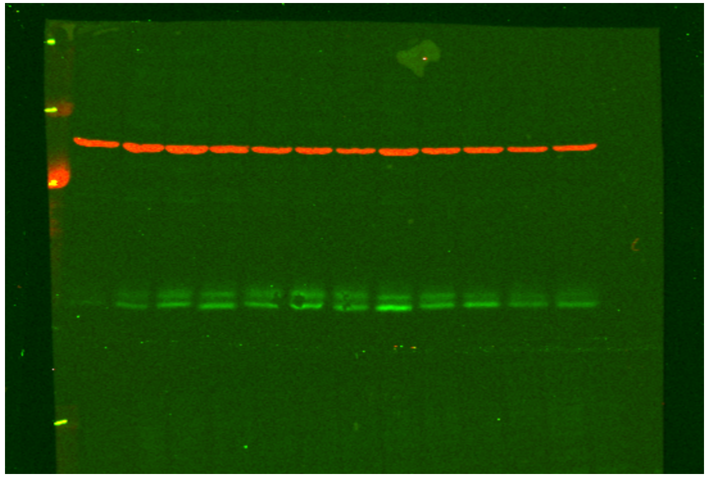

Supplementary Figure 3E

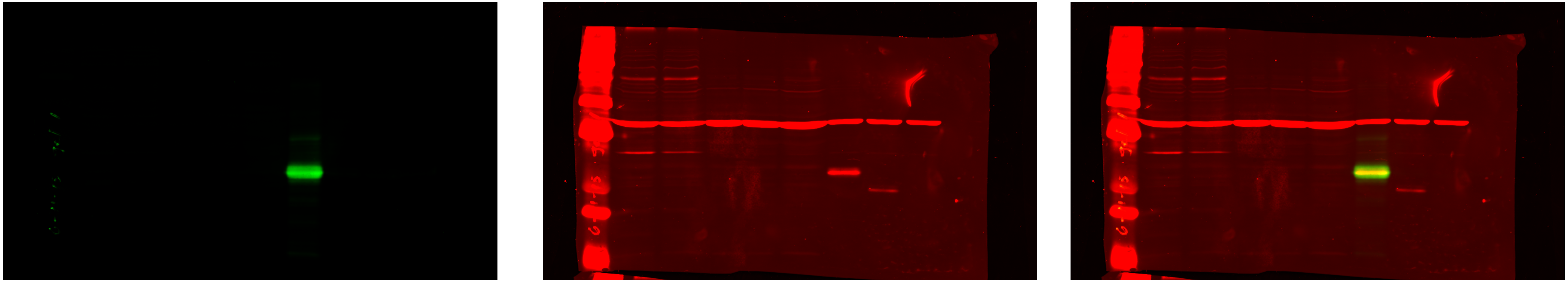

Supplement: Supplementary file 1 [file Data_Sheet_1.pdf]
